# Accuracy of a Novel Non-Invasive technology based EZSCAN system for the diagnosis of diabetes mellitus in Chinese

**DOI:** 10.1186/1758-5996-3-36

**Published:** 2011-12-22

**Authors:** Chang-Sheng Sheng, Wei-Fang Zeng, Qi-Fang Huang, Jean-Paul Deslypere, Yan Li, Ji-Guang Wang

**Affiliations:** 1 Centre for Epidemiological Studies and Clinical Trials, The Shanghai Institute of Hypertension, Ruijin Hospital, Shanghai Jiaotong University School of Medicine, Shanghai, China; 2 Impeto Medical, Paris, France

**Keywords:** EZSCAN, Diabetes mellitus, Iontophoresis technology

## Abstract

**Background:**

A new simple technique based on iontophoresis technology (EZSCAN, Impeto Medical, Paris, France) has recently been developed for the screening of diabetes. In the present study, we investigated the accuracy of this system for the diagnosis of diabetes mellitus in Chinese.

**Methods:**

We performed the EZSCAN test in diabetic and non-diabetic subjects. EZSCAN measures electrochemical conductance (EC) at forehead, hands and feet, and derives a diabetes index with a value ranging from 0 to 100. Diabetes mellitus was defined as a plasma glucose concentration of at least 7 mmol/l at fasting or 11.1 mmol/l at 2 hours after glucose load, or as the use of antidiabetic drugs.

**Results:**

The 195 study participants (51% men, mean age 52 years) included 75 diabetic patients (use of antidiabetic drugs 81%) and 120 non-diabetic subjects. EC (micro Siemens, μSi) was significantly (*P *< 0.001) lower in diabetic patients at the hands (44 vs. 61) and feet (51 vs. 69) locations, but not at the forehead (15 vs. 17, *P *= 0.39). When a diabetes index of 40 (suggested by the manufacturer) was used as the threshold, the sensitivity and specificity for the diagnosis of diabetes mellitus was 85% and 64%, respectively. In 80 patients who underwent an oral glucose tolerance test, EC at hands and feet and the diabetes index were significantly (*P *< 0.001) associated with both 2-hour post-load plasma glucose and serum glycosylated haemoglobin.

**Conclusions:**

EZSCAN might be useful in screening diabetes mellitus with reasonable sensitivity and specificity.

## Background

The prevalence of type 2 diabetes mellitus is high in most countries. According to the most recent nationwide survey in China, the prevalence of diabetes mellitus and impaired glucose metabolism was 9.7% and 15.5%, respectively [1]. While diabetes mellitus is becoming a major public health problem, the identification of this disease is still difficult [2]. The oral glucose tolerance test (OGTT) is complicated. The measurement of glycosylated haemoglobin (HbA1c) requires high cost, and has low sensitivity, and no fully standardized analytical method. The fasting blood glucose measurement either via venous or capillary sampling is currently used for screening, but is not sufficiently sensitive. Indeed, if fasting blood glucose was used solely in the diagnosis of diabetes mellitus, the sensitivity was as low as 40% [3].

The recently developed EZSCAN system employs a novel technology of reverse iontophoresis [4] and measures function of sweat glands as a parameter of small nerve fiber dysfunction that may present early in prediabetes [5,6]. The original measurement of the system is sweat chloride concentration, while administering a low-voltage electric current at the feet, hands and forehead. With an algorithm accounting for sex, age, body mass index, and systolic blood pressure, an index could be derived for the diagnosis of diabetes mellitus, impaired glucose metabolism, and diabetic neuropathy. The system has been tested in Europe [7] and India [8] for the diagnostic accuracy of diabetes mellitus. In the present Chinese study, we first investigated the accuracy of the derived diabetes index in the diagnosis of diabetes mellitus, and then correlated this index and the original electrochemical conductance measurement with concentrations of 2-hour post-load plasma glucose and serum HbA1c.

## Methods

### Study subjects

The study participants (n = 195) were recruited from known diabetic patients enrolled in a trial of self blood glucose monitoring (n = 54), from hypertensive patients hospitalized in the Department of Hypertension, Ruijin Hospital (Shanghai, China, n = 96), and from apparently healthy volunteers (n = 45). We performed the EZSCAN test in all these participants. In the 96 hospitalized hypertensive patients, we also performed OGTT and measured serum HbA1c concentration in those who had no history of diabetes mellitus (n = 80) after exclusion of 12 patients with known diabetes mellitus and 4 patients who declined to participate. The Ethnics Committee of Ruijin Hospital, Shanghai Jiaotong University School of Medicine (Shanghai, China) approved the study protocol. All subjects gave written informed consent.

### The EZSCAN test

The EZSCAN system is designed to evaluate sweat-gland function by measuring sweat chloride concentrations using reverse iontophoresis and chronoamperometry. Two sets of large-area nickel electrodes are used as anode and cathode. A direct current at an incremental voltage of 4 V or less is applied to the anode. This applied current generates voltage to the cathode and a current between the anode and cathode. This generated current is proportional to chloride concentration and measurable by chronoamperometry. The electrochemical conductance (μSi) is calculated as the ratio of the current measured over the constant power applied for the forehead (left and right parts), hands (left and right), and feet (left and right) and for the whole body. A diabetes index is then derived from these electrochemical conductance measurements with an algorithm accounting for sex, age, body mass index, and systolic blood pressure, for the classification of glucose metabolism as the presence (diabetes index ≥40) or absence of diabetes mellitus (diabetes index < 40).

A trained physician (CSS) performed the EZSCAN test, after he administered a questionnaire to collect information on medical history and the use of medications, and measured body weight and body height. Blood pressure was measured three times consecutively after the subjects had rested for at least 5 minutes in the sitting position. These three blood pressure readings were averaged for analysis. Before the EZSCAN test, the operator had to enter the following variables into the system: sex, age, body height, body weight, and systolic blood pressure, but the subject did not require for any preparation, such as fasting or no vigorous exercise. During the 2-min test, the subject was asked to stand up still with the hands and feet on the pads of electrode and with the headband on the forehead.

### OGTT and laboratory methods

A standard OGTT was performed after overnight fasting of 8-10 h according to the WHO recommendations [9] in subjects without known diabetes mellitus. Blood samples were drawn immediately and 30, 60, 120, and 180 minutes after an oral load of 75 g anhydrous glucose. Plasma glucose was measured by the glucose oxidase peroxidase method. Diabetes mellitus was defined as a plasma glucose of at least 7.0 mmol/L fasting or 11.1 mmol/L at 2 hours after glucose load, or as the use of antidiabetic agents [9]. HbA1c was measured by the HPLC method [10].

### Statistical methods

For database management and statistical analysis, we used SAS software (version 9.13, SAS Institute, Cary, NC, USA). Means and proportions were compared with the Student's *t*-test and Fisher's exact test, respectively. The Receiver Operating Characteristic (ROC) [11] curve was used to show sensitivity and specificity of the EZSCAN diabetes index for the diagnosis of diabetes mellitus. We calculated Pearson correlation coefficients to test the correlations of the original electrochemical conductance measurements and the derived diabetes index with concentrations of 2-hour post-load plasma glucose and serum HbA1c.

## Results

195 participants included 99 (51%) men and 75 (39%) diabetic patients, of whom 61 (81%) took antidiabetic medication. Diabetic patients, compared with non-diabetic subjects, were older (+14 years, *P *< 0.0001), and had a greater body mass index (+2 kg/m^2^, *P *= 0.0001) and higher systolic blood pressure (+9 mmHg, *P *= 0.003) and fasting plasma glucose (+2 mmol/L, Table 1).

**Table 1:** Characteristics of the study participants

Characteristic	Diabetic patients* (n = 75)	Non-diabetic subjects (n = 120)	*P *value
Men, n (%)	34 (45.3)	65 (54.2)	0.23
Age, years	60.5 ± 8.4	47.0 ± 16.2	< 0.0001
Body height, cm	162.7 ± 9.3	166.4 ± 9.1	0.006
Body weight, kg	70.4 ± 12.7	68.3 ± 12.9	0.26
Body mass index, kg/m^2^	26.6 ± 3.9	24.5 ± 3.3	0.0001
Systolic blood pressure, mm Hg	143.5 ± 14.2	136.4 ± 18.5	0.003
Diastolic blood pressure, mm Hg	80.2 ± 9.2	81.8 ± 10.2	0.50
Fasting blood glucose, mmol/L	6.86 ± 1.23	5.07 ± 0.59	< 0.0001
Use of anti-diabetic drugs, n (%)	61 (81.3%)	0	-

Values are mean ± standard deviation, or number of subjects (%).

*Diabetes mellitus was defined as a plasma glucose of at least 7.0 mmol/L fasting or 11.1 mmol/L at 2 hours post-load, or as the use of antidiabetic agents.

Diabetic patients, compared with non-diabetic subjects, had significantly (*P *< 0.0001) lower electrochemical conductance for the hands (44 vs. 61 μSi), feet (51 vs. 69 μSi), and the whole body (37 vs. 49 μSi) but not for the forehead (15 vs. 17 μSi, *P *= 0.39), and had significantly (*P *< 0.0001) higher EZSCAN diabetes index (67 vs. 35, Table 2).

**Table 2:** The electrochemical conductance and the EZSCAN diabetes index in diabetic and non-diabetic subjects

	Diabetic patients* (n = 75)	Non-diabetic subjects (n = 120)	Relative difference (95% confidence interval)	*P *value
Electrochemical conductance, μSi				
Feet	50.9 ± 21.9	69.1 ± 16.8	-26.3% (-18.4--34.3%)	< 0.0001
Hands	43.7 ± 20.4	61.2 ± 15.5	-28.6% (-20.3--36.9%)	< 0.0001
Forehead	15.1 ± 17.8	17.4 ± 18.8	-13.2% (-44.3-17.8%)	0.39
Overall	36.6 ± 15.8	49.2 ± 11.8	-25.8% (-17.7--33.7%)	< 0.0001
EZSCAN diabetes index (0-100)				
Mean ± SD	66.6 ± 25.8	34.6 ± 27.9	92.5% (70.0-115.6%)	< 0.0001
Median (inter-quartile range)	68 (48)	29 (47)	-	< 0.0001

Values are mean ± standard deviation, unless otherwise indicated.

*Diabetes mellitus was defined as a plasma glucose of at least 7.0 mmol/L fasting or 11.1 mmol/L at 2-hour post-load, or as the use of antidiabetic agents.

**Figure 1:**
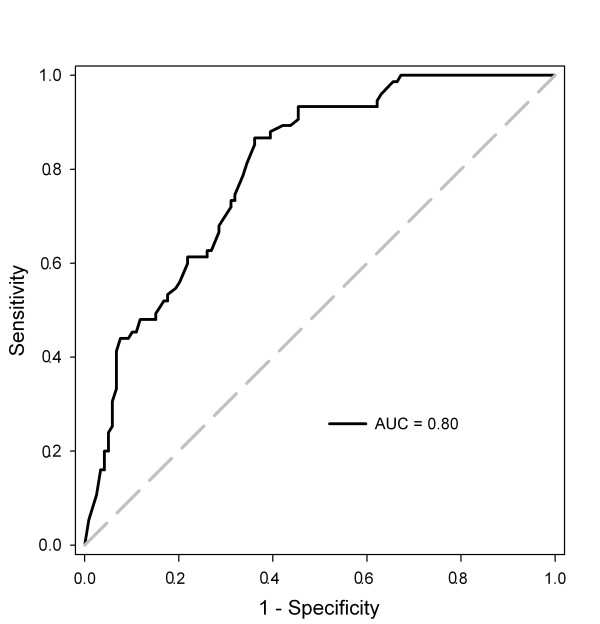
**Accuracy of EZSCAN for the diagnosis of diabetes mellitus, analyzed by Receiver Operating Characteristic (ROC) curve**.

The ROC curve shows the diagnostic accuracy of the derived EZSCAN diabetes index for the diagnosis of diabetes mellitus (Figure 1). The area under the curve (AUC) was 80% of the total square. If the EZSCAN diabetes index of 40 was used as the threshold for the diagnosis of diabetes mellitus in all subjects, the sensitivity and specificity were 85% and 64%, respectively. If the threshold increased to 50 and 60, the diagnostic sensitivity dropped to 67% and 60%, respectively, and the diagnostic specificity rose to 72% and 78%, respectively (Table 3). In 80 patients who had no history of diabetes mellitus and had an OGTT, 9 (11.3%) were diagnosed as diabetes mellitus by OGTT. In these patients, the EZSCAN diabetes index of 40 had higher sensitivity (78%) than fasting plasma glucose (44%) or serum HbA1c (56%, Table 3).

**Table 3:** Sensitivity and specificity for the diagnosis of diabetes mellitus

	Sensitivity (%)	Specificity (%)
EZSCAN in all subjects (n = 195)*		
EZSCAN diabetes index ≥ 40	85	64
EZSCAN diabetes index ≥ 50	67	72
EZSCAN diabetes index ≥ 60	60	78
EZSCAN in subgroup analysis (diabetes index ≥40 as threshold)		
Men (n = 99)	82	65
Women (n = 96)	87	63
< 54 years (median age, n = 100)	61	83
≥54 years (median age, n = 95)	93	24
Comparison of 3 methods in patients who had an oral glucose tolerance test (n = 80)*		
EZSCAN diabetes index ≥ 40	78	67
Fasting plasma glucose ≥ 7.0 mmol/L	44	100
Serum glycosylated haemoglobin ≥ 6.5%	56	93

*Diabetes mellitus was defined as a plasma glucose of at least 7.0 mmol/L fasting or 11.1 mmol/L at 2 hours post-load, or as the use of antidiabetic agents in all subjects (75 diabetic patients), and as a plasma glucose of at least 7.0 mmol/L fasting or 11.1 mmol/L at 2 hours post-load in patients who had an oral glucose tolerance test (9 diabetic patients).

After stratification for sex and age (below or above the median of 54 years), we calculated sensitivity and specificity of the diabetes index of 40 in men (n = 99) and women (n = 96) and in the younger (n = 100) and older (n = 95) subjects. The EZSCAN system had similar sensitivity and specificity in both sexes (*P*≥0.50), but tended to have higher sensitivity and lower specificity in older than younger subjects (*P*≤0.003, Table 3).

**Figure 2:**
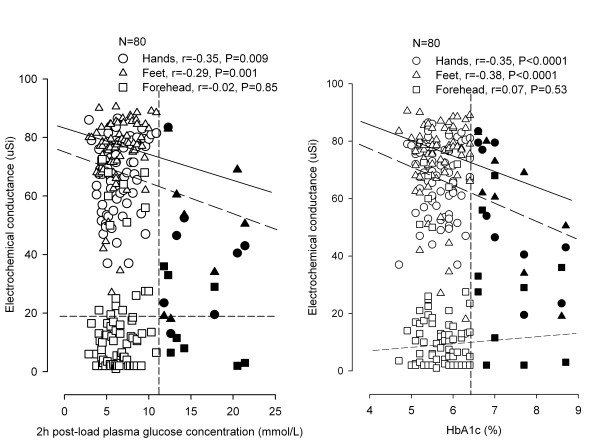
**Scatter plots with regression line for the relation of the electrochemical conductance at the hands (circles), feet (triangles) and forehead (squares) locations with 2-hour post-load plasma glucose concentration (left panel) and serum glycosylated haemoglobin (HbA1c, right panel) in patients who had an oral glucose tolerance test (n = 80)**. The dashed vertical line denotes the threshold for the diagnosis of diabetes mellitus. Open and closed symbols represent diabetic patients and non-diabetic subjects, respectively. Correlation coefficients and P values are given for the feet, hands and forehead locations separately.

**Figure 3:**
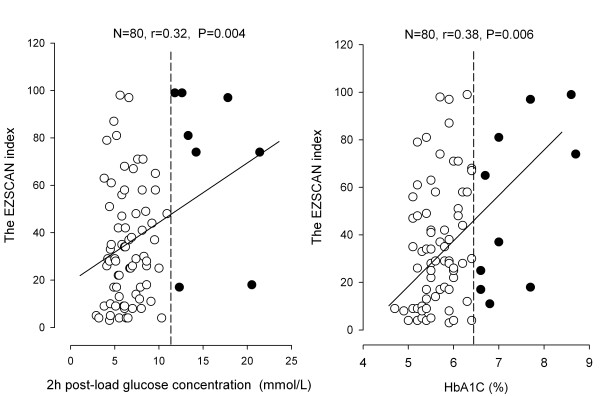
**Scatter plots with regression line for the relation of the EZSCAN diabetes index with 2-hour post-load plasma glucose concentration (left panel) and serum glycosylated haemoglobin (HbA1c, right panel) in patients who had an oral glucose tolerance test (n = 80)**. For further information, see legend to Figure 2.

In further analysis, we correlated the original electrochemical conductance measurement and the derived EZSCAN diabetes index with concentrations of 2-hour post-load plasma glucose and serum HbA1c in 80 patients who had an OGTT. The electrochemical conductance was significantly (*P*≤0.009) and negatively correlated with both 2-hour post-load plasma glucose concentration and serum HbA1c at the hands (r = -0.35 and r = -0.35, respectively) and feet (r = -0.29 and r = -0.38, respectively) but not (*P*≥ 0.53) at the forehead (r = -0.02 and r = 0.07, respectively, Figure 2). The EZSCAN diabetes index was significantly (*P*≤0.004) correlated with 2-hour post-load glucose (r = 0.32) and HbA1C (r = 0.38, Figure 3).

## Discussion

Our study demonstrated that EZSCAN, as a screening tool, had an acceptable accuracy for the diagnosis of diabetes mellitus. Indeed, if a diabetes index of 40 would be used as the threshold, the diagnostic sensitivity and specificity was 85% and 64%, respectively. In addition, the electrochemical conductance at the hands and feet but not at the forehead was significantly associated with 2-hour post-load plasma glucose concentration and serum HbA1C concentration.

To the best of our knowledge, only 2 published studies investigated the accuracy of EZSCAN for the diagnosis of impaired glucose metabolism. In 212 subjects recruited in India [7], EZSCAN, when a diabetes index of 50 was used as the threshold, had a sensitivity of 75% for the diagnosis of diabetes mellitus (n = 24), and a specificity of 54% in patients with normal glucose tolerance and without the metabolic syndrome (n = 101). The corresponding sensitivity and specificity for fasting plasma glucose (≥ 7.0 mmol/L) were 29% and 99%, respectively. In Indians, EZSCAN apparently had higher sensitivity but lower specificity than fasting glucose. In 133 diabetic patients and 41 healthy volunteers recruited in France [8], EZSCAN had a sensitivity of 75% and a specificity of 100%.

Taken the results of the 2 previous studies and our research together, EZSCAN seemed to have consistent and constant sensitivity (75% in Indians and French and 85% in Chinese) but divergent and variable specificity (54%, 100%, and 64%, respectively) across populations. The heterogeneous specificity might be attributable to the differences in characteristics of participants between these 3 studies, because subjects who had any risk factor for diabetes mellitus (≥ 45 years, infrequent physical activity, and first-degree relative with diabetes) were excluded from the non-diabetic control group of the French study [8] but not the Indian [7] and our Chinese studies. Nonetheless, it is also possible that the algorithm for the computation of the diabetes index and the cutoff value for the diagnosis of diabetes mellitus generated from the French population cannot directly be generalized to populations of other ethnicities.

Despite different diagnostic accuracy across populations, EZSCAN does measure something that reflects or is related to glucose metabolism. The original electrochemical conductance measurement at hands and feet was different between diabetic and non-diabetic subjects, and associated with 2-hour post-load plasma glucose and with serum HbA1c. The EZSCAN diabetes index was also associated with 2-hour post load plasma glucose and serum HbA1c.

Our finding that the electrochemical conductance was significantly different between diabetic and non-diabetic subjects only at the feet and hands locations but not at forehead are in keeping with the results of the French study [8], and might have important implications for future upgrading of the EZSCAN system. In the French study, diabetic patients, compared with non-diabetic subjects, had a lower electrochemical conductance at the feet and hands locations but an even higher value at forehead [8]. The electrochemical conductance measures function of sweat glands and activity of the corresponding autonomic nerves that are longest at the feet, intermediate at the hands, and shortest at the forehead [12]. We speculate that longer nerves are more susceptible for neuropathic lesions, regardless as a complication or in reverse as a cause of diabetic mellitus [13-15]. Thus, only the electrochemical conductance at the feet and hands locations is sufficiently sensitive to identify the presence of diabetes mellitus, and should be used in the upgraded system.

One of the major strengths of our study was that we performed OGTT and measured HbA1c, and compared the accuracy of the EZSCAN system with that of fasting plasma glucose and HbA1c. Nonetheless, our study had a small sample size and a cross-sectional design. A larger prospective study is apparently required.

In conclusion, EZSCAN is accurate in the diagnosis of diabetes mellitus with reasonable sensitivity and specificity as a screening tool. With the increasing use and knowledge, EZSCAN might bring revolutionized changes to the identification of diabetes mellitus as a screening tool and also to the understanding of mechanisms of blood glucose regulation as a research tool. More research is apparently required to investigate the significance of EZSCAN for risk stratification and prediction of cardiovascular and renal events in prospective studies, and to explore the role of EZSCAN in therapeutic monitoring of diabetes mellitus in controlled clinical trials. Before the evidence is becoming available, this system should be recommended for the use in the research setting.

## Competing interests

The authors declare that they have no competing interests.

## Authors' contributions

CSS carried out the field work, performed statistical analysis, and together with JGW drafted the manuscript. WFZ and QFH participated in the field work. JPD provided valuable help on the use of the EZSCAN system. YL and JGW conceived of, designed and coordinated the study. All authors read and approved the final manuscript.
